# Genome Sequence of *Prosthecochloris* sp. Strain CIB 2401 of the Phylum *Chlorobi*

**DOI:** 10.1128/genomeA.01222-16

**Published:** 2016-11-03

**Authors:** Shaza Nabhan, Boyke Bunk, Cathrin Spröer, Zhenfeng Liu, Donald A. Bryant, Jörg Overmann

**Affiliations:** a Leibniz Institute DSMZ-German Collection of Microorganisms and Cell Cultures, Braunschweig, Germany; b Department of Biochemistry and Molecular Biology, The Pennsylvania State University, University Park, Pennsylvania, USA

## Abstract

To date, only 13 genomes of green sulfur bacteria (family *Chlorobiaceae*) have been sequenced. The sequenced strains do not cover the full phylogenetic diversity of the family. We determined the complete genome sequence of *Prosthecochloris* sp. strain CIB 2401, thereby increasing the genome information for the poorly represented marine *Chlorobiaceae*.

## GENOME ANNOUNCEMENT

*Prosthecochloris* sp. strain CIB 2401, previously named *Pelodictyon phaeum* CIB 2401, is a green sulfur bacterium of the family *Chlorobacteriaceae* (1). This family currently comprises the three genera, *Chlorobium*, *Chlorobaculum*, and *Prosthecochloris* (2), of which the genus *Prosthecochloris* is typically marine (3). *Prosthecochloris* sp. CIB 2401 was isolated from the hypersaline Cibollar Lake, a coastal brackish lagoon on Mallorca Island (4, 5).

For genome sequencing, SMRTbell template libraries were prepared according to the instructions from Pacific Biosciences (PacBio, Menlo Park, CA, USA), according to the procedure and checklist for 10-kb template preparation and sequencing. About 8 µg of genomic DNA was sheared using g-Tubes from Covaris, Woburn, MA, USA. DNA was concentrated, end repaired, and ligated to hairpin adapters using the DNA/polymerase binding kit 2.0 from PacBio. The SMRTbell template was treated with exonuclease to remove incomplete reaction products. The conditions for annealing of sequencing primers and binding of polymerase to the purified template were assessed with the Calculator in RS Remote (PacBio). Two single-molecule real-time (SMRT) cells were sequenced using the PacBio RSII with a 90-min movie for each library. The *Prosthecochloris* sp. CIB 2401 genome was assembled employing the RS_HGAP_Assembly_Advanced.1 protocol (SMRT Portal version 2.0.0) based on 75,813 reads. The following parameters were chosen. For filtering: minimum subread length, 500; minimum polymerase read quality, 0.75; and minimum polymerase read length, 500. For assembly: compute minimum seed read length, true. For BLASR options: minReadLength, 200; maxScore, 1,000; bestn, 24; maxLCPLength, 16; nCandidates, 100; allow partial alignments, true; Trim FASTQ output, true; TrimOpts qvCut, 30; minSeqLen, 500; use CCS, false; target coverage, 15; overlapper error rate, 0.06; overlapper min length, 40; and overlapper k-mer, 14. A single contig for the consensus assembly was obtained. A manual check of the assemblies was performed using SMRT View and IGV (6). The contig was trimmed, circularized, and adjusted to *dnaA* as the first gene. Resequencing with the BridgeMapper protocol was done twice on the circular chromosome of *Prosthecochloris* sp. CIB 2401, A finishing quality of 100% (QV 60) was estimated.

The genome size of *Prosthecochloris* sp. CIB 2401 is 2,399,849 bp, and the G+C content is 52.13%. Annotation was carried out by Prokka and yielded a total of 2,200 coding sequences (CDSs), which makes 91.6% coding percentage. An open reading frame of 52,034 bp represents the largest putative coding sequence in the genome. Hence, it is the third largest CDS present in a green sulfur bacterium (GSB) genome after Cag_0614 and Cag_0616 of *Chlorobium chlorochromatii* CaD3 (7). The *Prosthecochloris* sp. CIB 2401 genome comprises two rRNA operons and 47 tRNAs and 3,122 domains of 1,399 Pfam, 16 of which are unique in comparison to other green sulfur bacteria sequences. Nine of the 10 genes encoding the chlorosome proteins (8) (*csmABCEFHIJX*) were found in the CIB 2401 genome. The gene marker *bciD* for the synthesis of bacteriochlorophyll *e* (9) is present. The genome possesses a gene cluster for gas vesicle synthesis (10). Of 38 cytochrome genes traced, *Prosthecochloris* sp. CIB 2401 harbors 18 genes, including the two subunits of the cytochrome *bd* (ubiquinol) oxidase *cydAB* (Ptc2401_01824 and Ptc2401_01825), but it lacks the gene cluster of cytochrome *cbb*_3_ oxidase.

### Accession number(s).

The nucleotide sequence has been deposited at NCBI GenBank under the accession no. CP016432.
